# Epidemiology of proximal and diaphyseal humeral fractures in children: an observational study from the Swedish Fracture Register

**DOI:** 10.1186/s12891-022-05042-0

**Published:** 2022-01-28

**Authors:** Sarah Daag Jacobsen, Richard Marsell, Olof Wolf, Yasmin D. Hailer

**Affiliations:** Department of Surgical Sciences, Section of Orthopaedics, Uppsala University, Uppsala, Sweden

**Keywords:** Epidemiology, Children, Humerus, Fracture, Treatment, Swedish fracture register

## Abstract

**Background:**

Most fractures in children are fractures of the upper extremity. Proximal and diaphyseal humeral fractures account for a minority of these fractures. To our knowledge, few previous reports address these fractures. This study aimed to describe the epidemiology and current treatment of proximal and diaphyseal humeral fractures by using the Swedish Fracture Register (SFR).

**Methods:**

In this nationwide observational study from the SFR we analysed data on patient characteristics, injury mechanism, fracture classification and treatment. We included patients aged < 16 years at time of injury with proximal or diaphyseal humeral fracture registered in 2015–2019.

**Results:**

1996 (1696 proximal and 300 diaphyseal) fractures were registered. Proximal fractures were more frequent in girls whereas diaphyseal fractures were more frequent in boys. The median age at fracture was 10 years in both fracture types but patient’s age was more widespread in diaphyseal fracture (IQR 5–13 compared to IQR 7–12 in proximal). In both sexes, the most registered injury mechanism was fall. Horse-riding was a common mechanism of injury in girls, whereas ice-skating and skiing were common mechanisms in boys. Most proximal fractures were metaphyseal fractures. Most diaphyseal fractures were simple transverse or oblique/spiral fractures. The majority of fractures were treated non-surgically (92% of proximal and 80% of diaphyseal fractures). The treatment method was not associated with the patient’s sex. Surgery was more often performed in adolescents. The most common surgical methods were K-wire and cerclage fixation in proximal fracture and intramedullary nailing in diaphyseal fracture.

**Conclusion:**

Following falls, we found sex-specific sport activities to cause most proximal and diaphyseal paediatric fractures. Further studies on prophylactic efforts in these activities are needed to investigate whether these fractures are preventable. The majority of the fractures were treated non-surgically, although surgical treatment increased with increasing age in both sexes.

**Trial registration:**

Not applicable. The present study is a register-based cohort study. No health care intervention had been undertaken*.*

## Background

One in three children sustain a fracture during childhood and adolescence [1]. Previous studies report that almost 80% of these fractures are fractures of the upper extremity [2, 3] and that humeral fractures account for less than 10% of all fractures in children [4, 5]. The majority (70%) of these paediatric humeral fractures are supracondylar fractures [3–7]. Most research on humeral fractures in children therefore addresses only distal humeral fractures. Proximal and diaphyseal fractures subsequently remain relatively unexplored. Most studies on proximal and diaphyseal humeral fractures are either single-center studies with a retrospective design and relatively few patients included [8] or focus solely on different treatment techniques [9].

Paediatric fractures in general have a 1:1.5 ratio between girls and boys, and a unimodal age distribution with an observed incidence peak in early teenage years [1, 4, 10]. Most fractures are caused by falls or sport activities and the incidence of sport-related injuries increases with age [4, 10]. Horse-related injuries are predominantly seen in girls and winter-related sports and transportation accidents in boys [4, 7, 11, 12]. It is unclear if this pattern applies to proximal and diaphyseal humeral fractures as well.

There are some population-based studies of fractures in the Scandinavian paediatric population [3, 4, 7, 11]. But to our knowledge, there is no published nationwide epidemiological study addressing humeral fractures other than distal fractures in a Scandinavian paediatric population.

This nationwide population-based observational study of proximal and diaphyseal humeral fractures in children and adolescents aims to describe the epidemiology of these fractures and possible associations between sex, age and the used treatment method. In addition, we aim to identify activities with high risk of injuries in the paediatric population, which in turn affects the patient inflow on orthopaedic care units as well as health economy.

Main hypotheses were that (i) proximal and diaphyseal humeral fractures are more common in boys, (ii) most injuries occur during recreational activities and (iii) that the incidence of surgical treatment increases with age. We hypothesised an increase in surgery with age due to the decrease in remodelling capacity with closed growth plates [13, 14].

## Methods

### Data collection and study population

This nationwide population-based observational study was conducted using the Swedish Fracture Register (SFR). The SFR is a national quality register compiling data on patient characteristics, injury mechanism, fracture classification and treatment. The treating physician is responsible for registration including classification of the fracture. The register was established in 2011 and since May 2015 the register has expanded to include also paediatric fractures [15]. Since 2021 all orthopaedic departments in Sweden report to the SFR (100% coverage). Comparing the registered paediatric humeral fractures in the SFR with the National Patient Register, the completeness was > 70% in half of the affiliated hospitals and varying between 10 and 70% in the remaining hospitals. Most of the latter only registered surgically treated fractures (internal audit, data not published).

All patients < 16 years of age at the time of injury, with a proximal or diaphyseal humeral fracture registered between 1 January 2015 and 31 December 2019, were included (8052 fractures). Supracondylar humeral fractures (6056 fractures) were excluded. Patients with two fractures (127 patients) registered within the time of study (either patients with multiple fractures and/or refractures) remained in the study.

### Variables

Variables of interest included: patient’s age, sex, injury date and injury mechanism. Furthermore, anatomical fracture classification and treatment method were assessed.

The mechanism of injury was divided into eight groups: simple falls, falls from height, unspecified falls, transportation accidents, stress/pathological/spontaneous fractures, non-accidental, other accidents and no mechanism registered (missing data). “Simple fall” included all falls in the same level (trips, in snow/ice, ice-skating/skiing, nudges) and “fall from height” all falls from another level (from furniture, playground facilities, stairs, trees, buildings). “Unspecified falls” were injuries registered as falls without information about which level they occurred in. “Transportation accidents” were divided into several subgroups; pedestrian, bicycle/motorcycle (including other small vehicles), car (including truck), horse-riding and unspecified transportation accidents. “Non-accidental injuries” were all injuries due to fights, abuse and self-destructive acts. “Other accidents” were injuries due to mechanical and living forces.

**Fig. 1:**
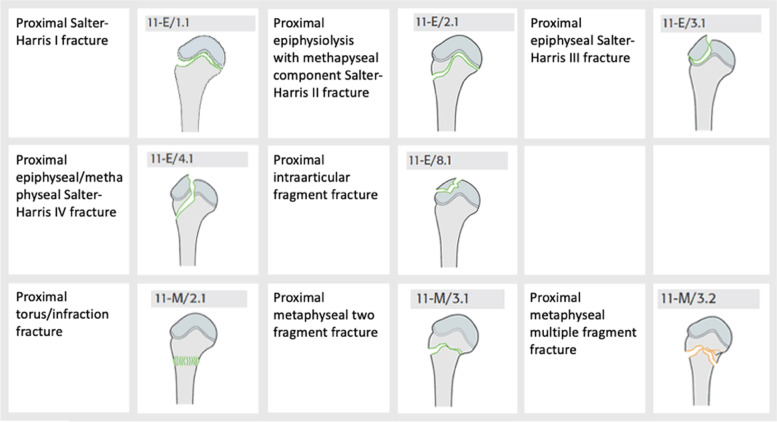
Fracture classification according to the AO/OTA classification system. From the Swedish Fracture Register

**Fig. 2:**
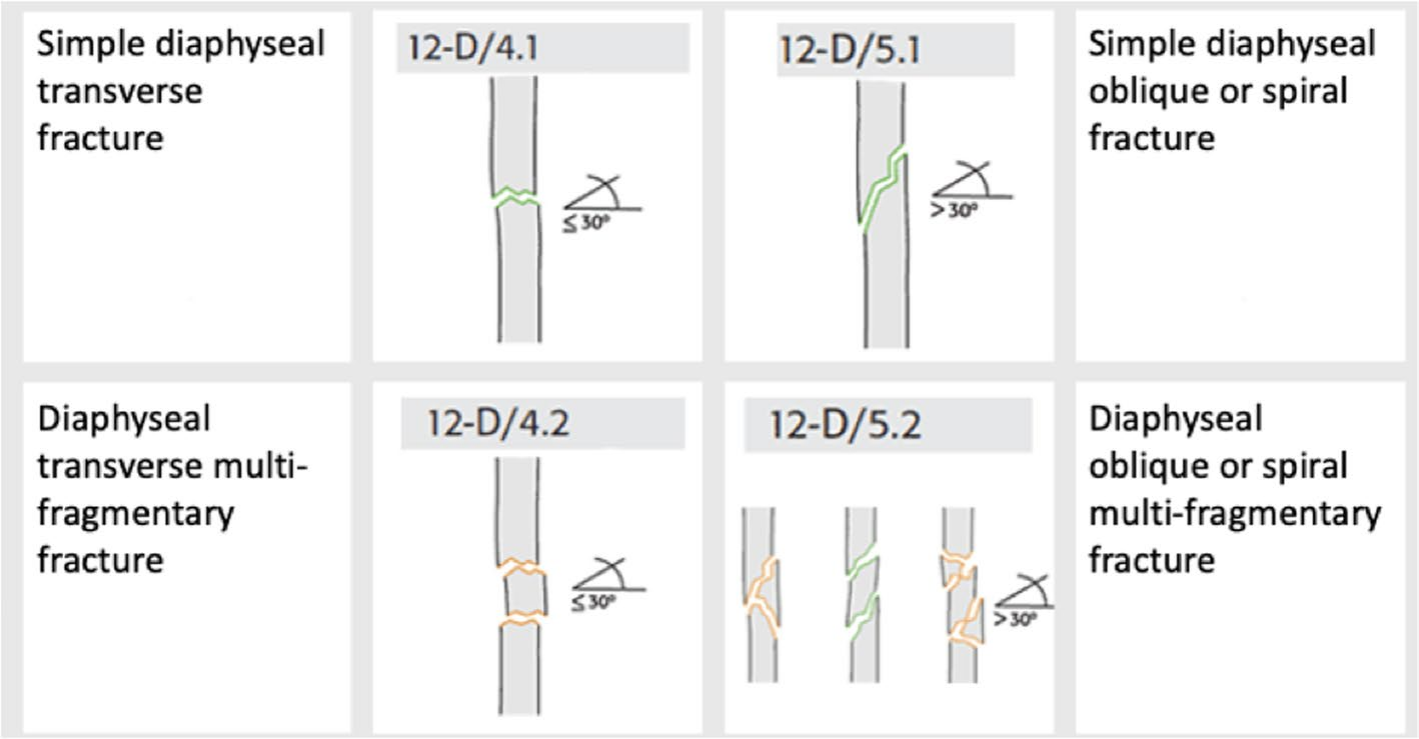
Fracture classification according to the AO/OTA classification system. From the Swedish Fracture Register

The fractures were categorized according to the paediatric AO/OTA classification system (Figs. 1 and 2). The fractures were also divided into groups of adult patients with closed growth plates or paediatric patients with open growth plates. In proximal fractures, fractures categorized as Salter-Harris III, IV, and intraarticular fragments were all categorized as “intraarticular fractures”.

Treatment was divided into two main categories; non-surgical and surgical treatment. The surgical treatment category also included those patients which had a change of their treatment regime from initial non-surgical treatment to surgical within 2 weeks of the injury. The non-surgical group was further divided into a non-surgical or non-surgical with closed reduction group, and the surgical group to; a) closed reduction under general anaesthesia, b) k-wire and cerclage, c) intramedullary nailing (including both rigid and flexible nails), d) plate fixation, e) screw fixation, f) combined osteosynthesis and g) external fixation groups. The chosen method of treatment was then surveyed in four different age groups; infancy and toddlerhood (0–3 years), preschool (4–6 years), school-age (7–12 years) and adolescence (13–15 years).

### Statistics

The statistical analyses were performed using Excel (Microsoft Excel for Mac version 16.31, Microsoft Corporation, Redmond, WA) and R version 3.6.3 (February 29, 2020). Descriptive statistics (counts, median with interquartile range and percentage) were used to describe the collected data. Logistic regression was performed to estimate the odds ratio (OR). Statistical significance was defined as *p* < 0.05.

## Results

1696 (21%) of all registered humeral fractures (8052) were proximal fractures, and 300 (4%) were diaphyseal. There were 5 open fractures, 1 (0.01%) in girls and 4 in boys (0.2%) (Table 1). Two patients (both boys) died within the study period. No data on their date or cause of death was available.

**Table 1:** Basic characteristics of all fractures

	Proximal	Diaphyseal	Total
**Sex**			
Girls, n (%)	1004 (59)	103 (34)	1107 (55)
Boys, n (%)	692 (41)	197 (66)	889 (45)
Total, n (%)	1696 (100)	300 (100)	1996 (100)
**Median age (IQR)**			
Girls	10 (7–12)	9 (5–12)	10 (7–12)
Boys	11 (8–13)	10 (6–13)	11 (7–13)
Total	10 (7–12)	10 (5–13)	11 (9–13)
**Open fractures**			
Girls, n (%)	0	1	1 (0.01)
Boys, n (%)	2	2	4 (0.2)
Total, n (%)	2 (0.1)	3 (1)	5 (0.3)

### Patient characteristics

**Fig. 3:**
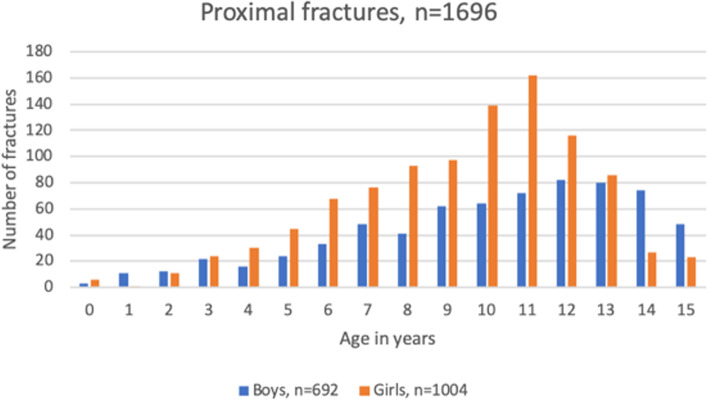
Distribution of age in patients (boys and girls) with proximal fracture

**Fig. 4:**
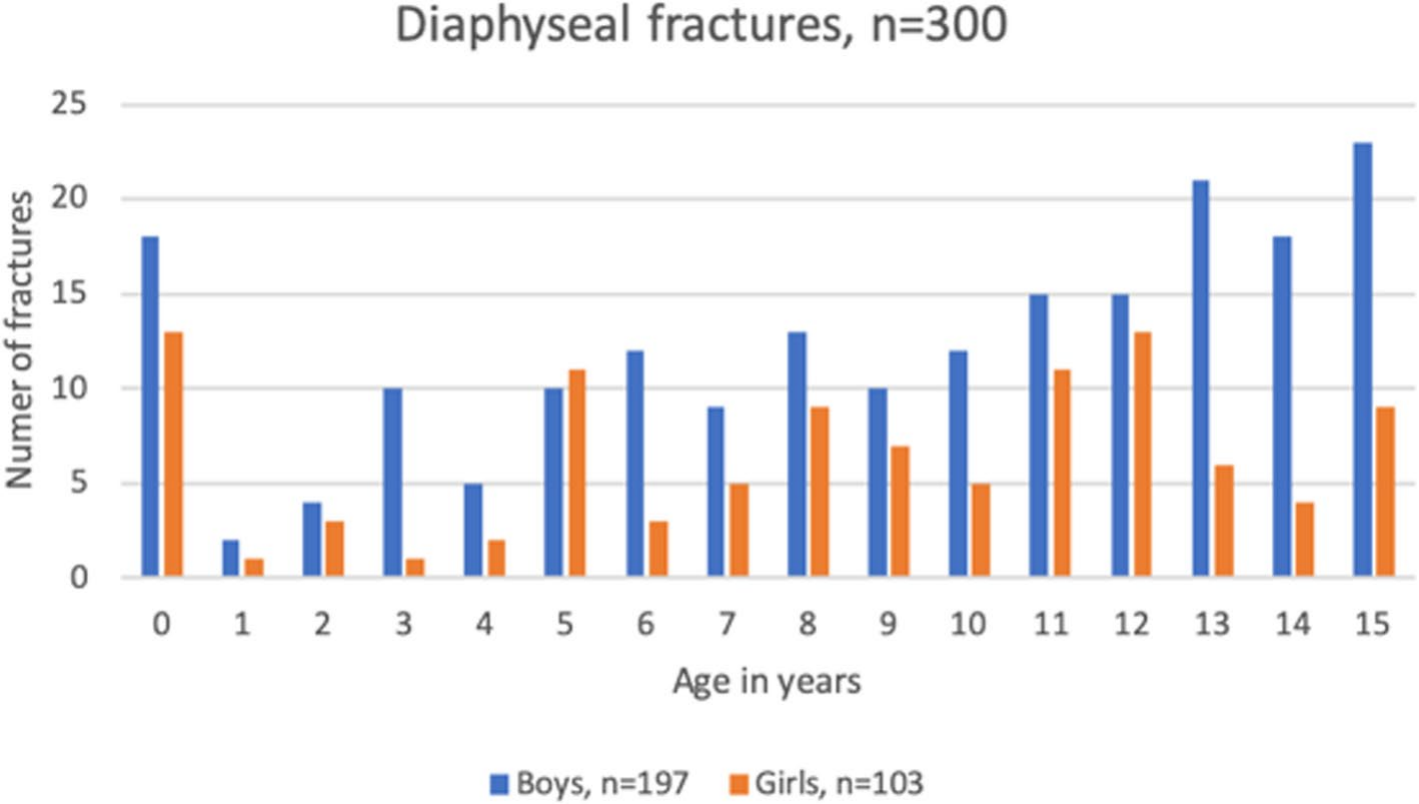
Distribution of age in patients (boys and girls) with diaphyseal fracture

The total girl:boy-ratio was 1.5:1 in proximal fractures and 1:1.9 in diaphyseal. The proximal to diaphyseal fracture ratio was 9.7:1 in girls and 3.5:1 in boys*.* In proximal fractures the age distribution was unimodal with a peak at 11 years in girls and a peak at 12 years in boys (Fig. 3). In diaphyseal fractures the age distribution was uneven (Fig. 4).

**Fig. 5:**
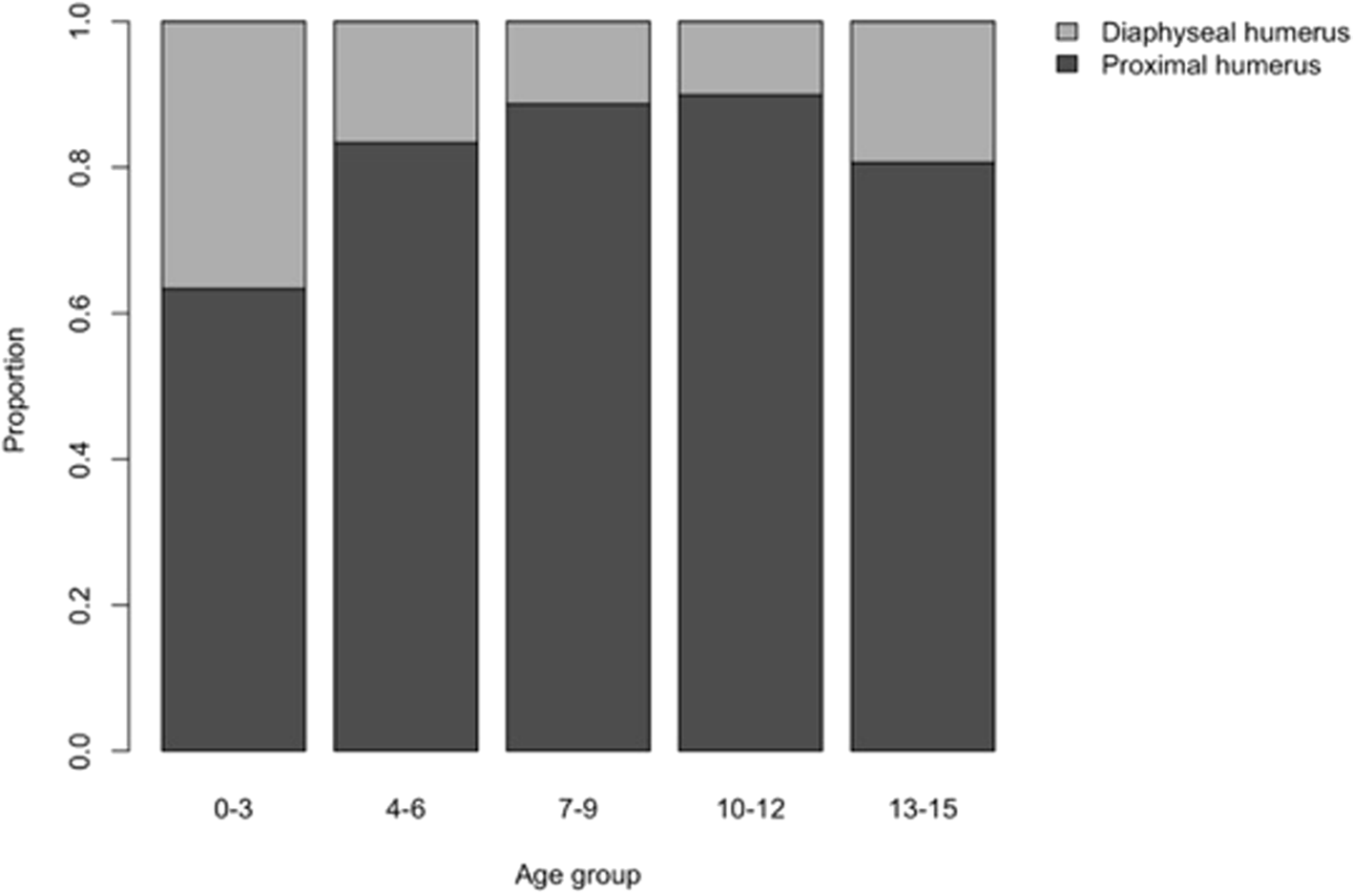
Proportion of fracture (proximal or diaphyseal) location by age group

Regarding the proportion between proximal and diaphyseal humeral fractures by age group, patients between 0 and 3 years had the highest percentage of diaphyseal fractures (Fig. 5).

### Seasonal variation and injury mechanism

**Fig. 6:**
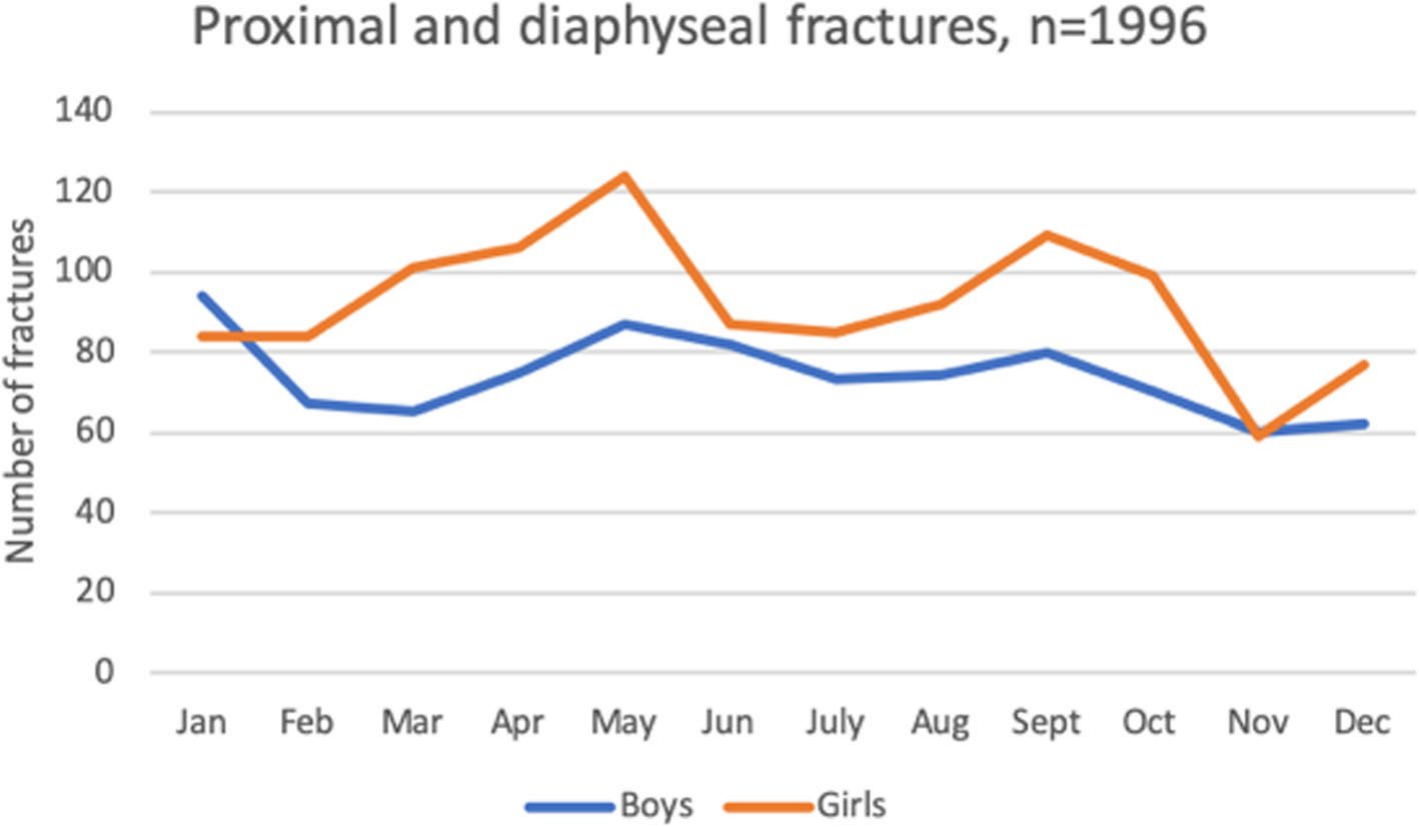
Seasonal variation of proximal and diaphyseal fractures for boys and girls respectively

Among girls, most fractures (124, 11%) were registered in May (Fig. 6). The dominating mechanisms of injury for these fractures were falls at playground facilities (28, 23%) and due to horse-riding (17, 14%). Among boys, most fractures (94, 11%) were registered in January. The dominating injury mechanism for these fractures were fall in the same level on snow/ice (19, 20%) or due to winter sport activities (19, 20%).

The overall dominating injury mechanism irrespective of the season were falls in both sexes. Injury details of the injury mechanisms are shown in Table 2.

**Table 2:** Injury mechanism for proximal and diaphyseal humeral fractures presented for girls, boys and total

	Proximal			Diaphyseal		
	Girls, n (%)	Boys, n (%)	Total, n (%)	Girls, n (%)	Boys, n (%)	Total, n (%)
**Simple falls**	287 (29)	258 (37)	545 (32)	19 (18)	56 (28)	75 (25)
Tripping	149	127	275	11	30	41
Ice-skating/skiing	70	65	135	5	14	19
On snow/ice	42	40	82	2	9	11
**Falls from height**	347 (35)	206 (30)	553 (33)	28 (27)	46 (23)	74 (25)
Playground facilites	169	115	284	19	26	45
**Unspecified falls**	78 (8)	34 (5)	112 (7)	3 (3)	10 (5)	13 (4)
**Transportation accidents**	204 (20)	118 (17)	322 (19)	24 (23)	25 (13)	49 (16)
Pedastrian	2	1	3	1	2	3
Bicycle/motorcycle	43	106	149	8	19	27
Car	1	1	2	1	3	4
Horse-riding	150	5	155	13	1	14
Unspecified	8	5	13	1	0	1
**Stress/pathological/spontaneous**	2 (0.2)	2 (0.3)	4 (0.2)	2 (2)	14 (7)	16 (5)
**Non-accidental**	3 (0.3)	3 (0.4)	6 (0.4)	3 (3)	4 (2)	7 (2)
**Other accidents**	60 (6)	50 (7)	110 (6)	17 (17)	36 (18)	53 (18)
**Not registered**	23 (2)	21 (3)	44 (3)	7 (7)	6 (3)	13 (4)
**Total**	1004 (100)	692 (100)	1696 (100)	103 (100)	197 (100)	300 (100)

Among girls, most transportation accidents were caused by horse-riding. Among boys, most transportation accidents were caused by riding a bike or moped.

### Fracture classification

**Fig. 7:**
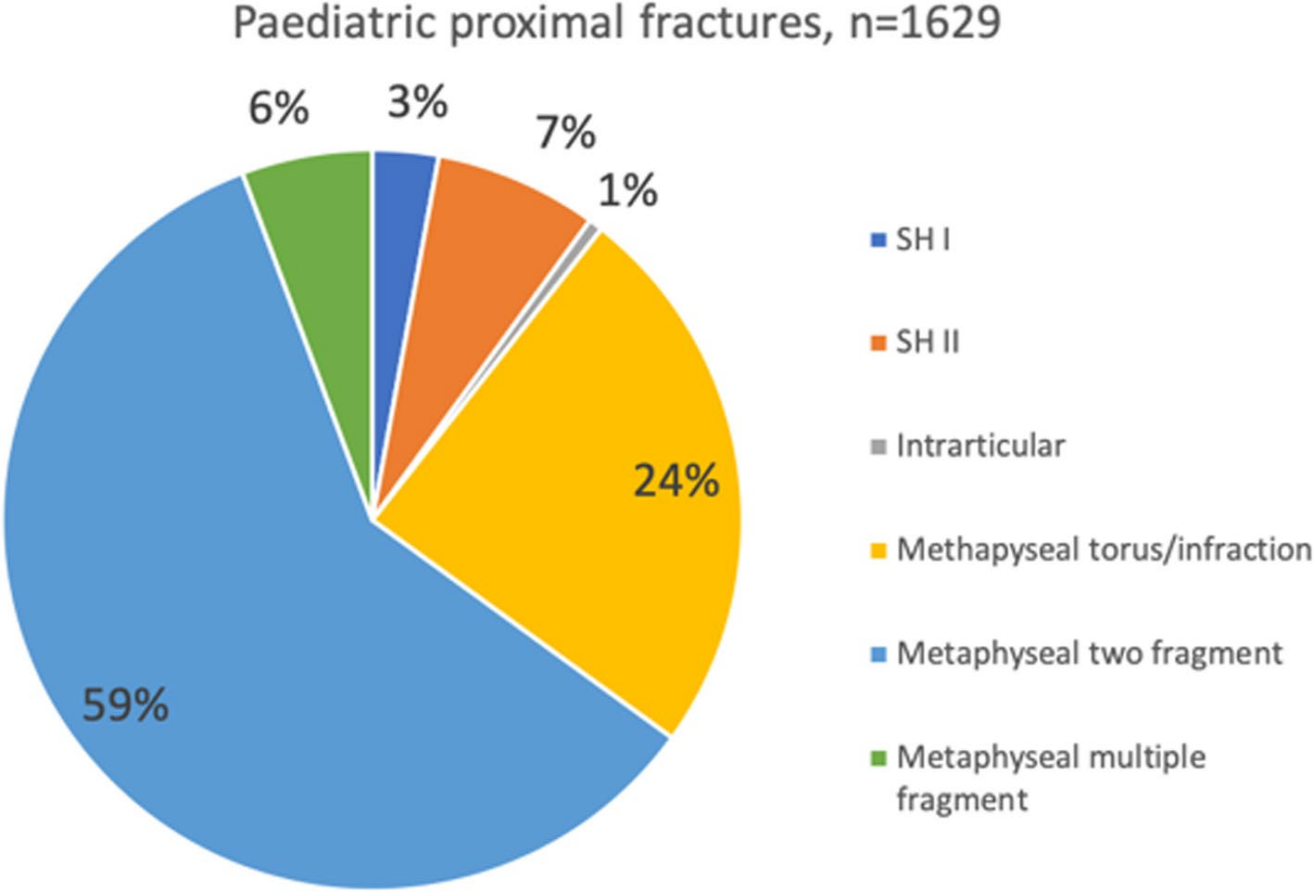
Fracture classification in paediatric proximal fractures according to AO/OTA classification

1696 (96%) of all proximal fractures were classified as paediatric fractures (open growth plates) and the majority of those were located in the metaphysis (Fig. 7). There was no difference in fracture type between girls and boys. Only 30 (3%) of the proximal fractures in girls and 22 (3%) in boys were classified as adult fractures (closed growth plates). In 15 fractures (0.3%) information about the growth plate status was missing.

**Fig. 8:**
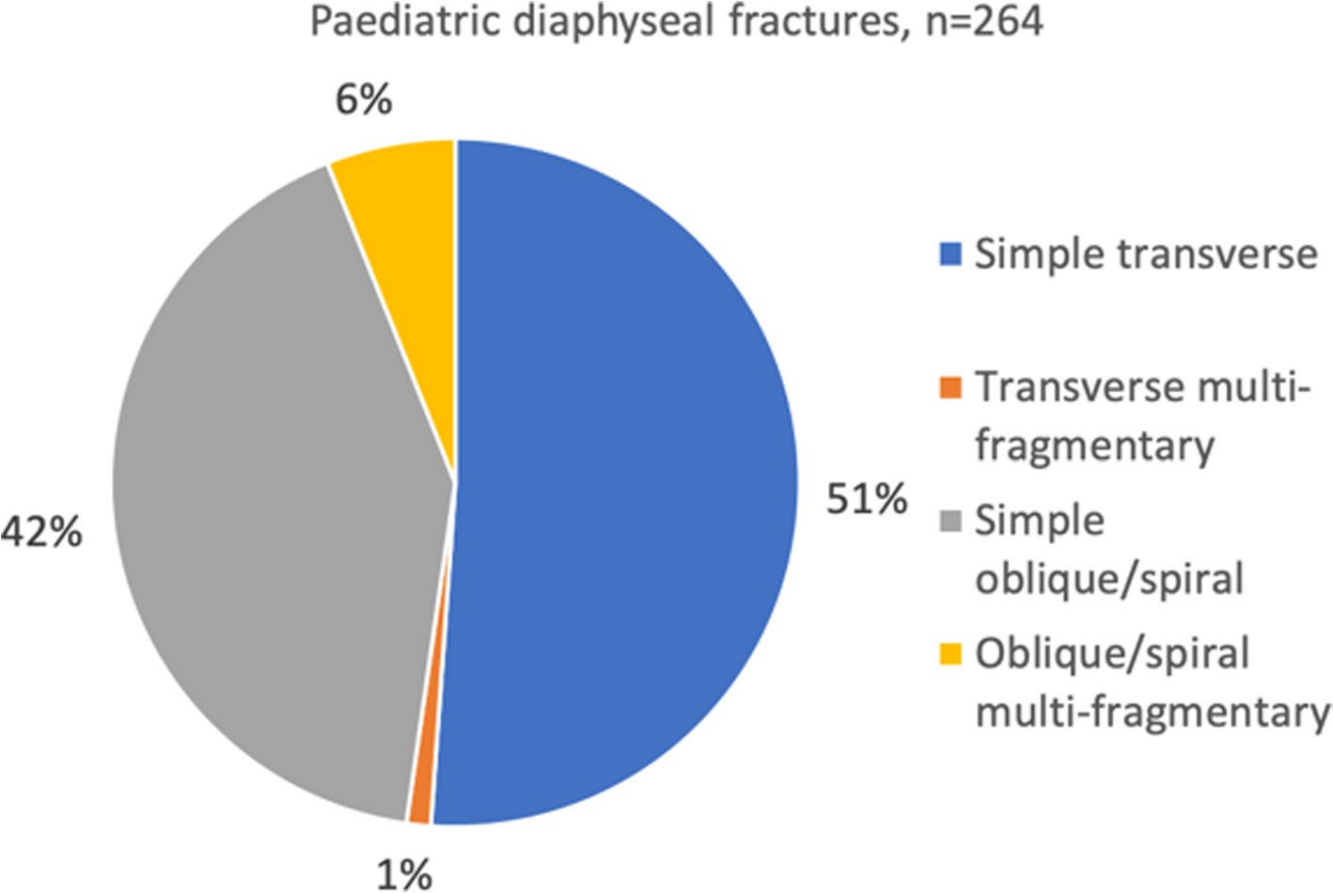
Fracture classification in paediatric diaphyseal fractures according to AO/OTA classification

300 (88%) of diaphyseal fractures were classified as paediatric fractures (open growth plate) and the majority of those were simple transverse or oblique/spiral (Fig. 8). There was no difference in boys and girls. Of the diaphyseal fractures in girls, 15 (15%) had closed growth plates and were therefore classified as adult fractures. This proportion was somewhat higher than in boys (8%). In 6 cases (0.2%) information about the growth plate status was missing.

### Treatment

The majority of proximal (1561, 92%) and diaphyseal (241, 80%) humeral fractures were treated non-surgically. Logistic regression analysis revealed increased surgical intervention with increasing age in both locations. There was no significant difference in odds for surgical treatment between the sexes, see Table 3.

**Table 3:** Odds ratios for surgical treatment in relation to age at injury and sex

Proximal humerus fracture	OR	95% Confidence intervall	***P***-value
Age at fracture	1.3	1.2–1.4	< 0.001
Sex (girl)	0.9	0.6–1.4	0.7
**Diaphyseal humerus fracture**	**OR**	**95% Confidence intervall**	***P*-value**
Age at fracture	1.2	1.1–1.3	< 0.001
Sex (girl)	0.9	0.4–1.8	0.7

**Fig. 9:**
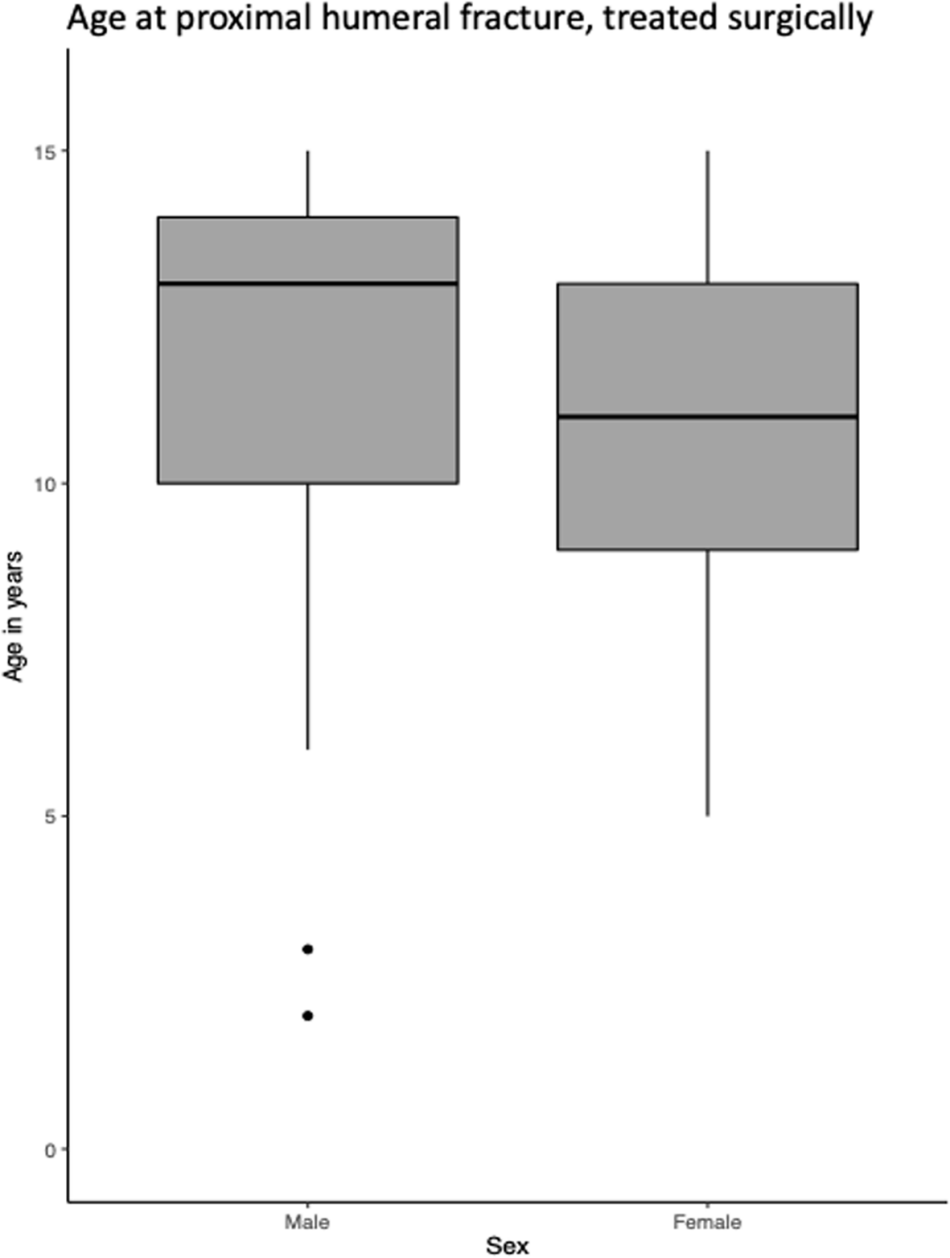
Age at surgical treated proximal humeral fracture

**Fig. 10:**
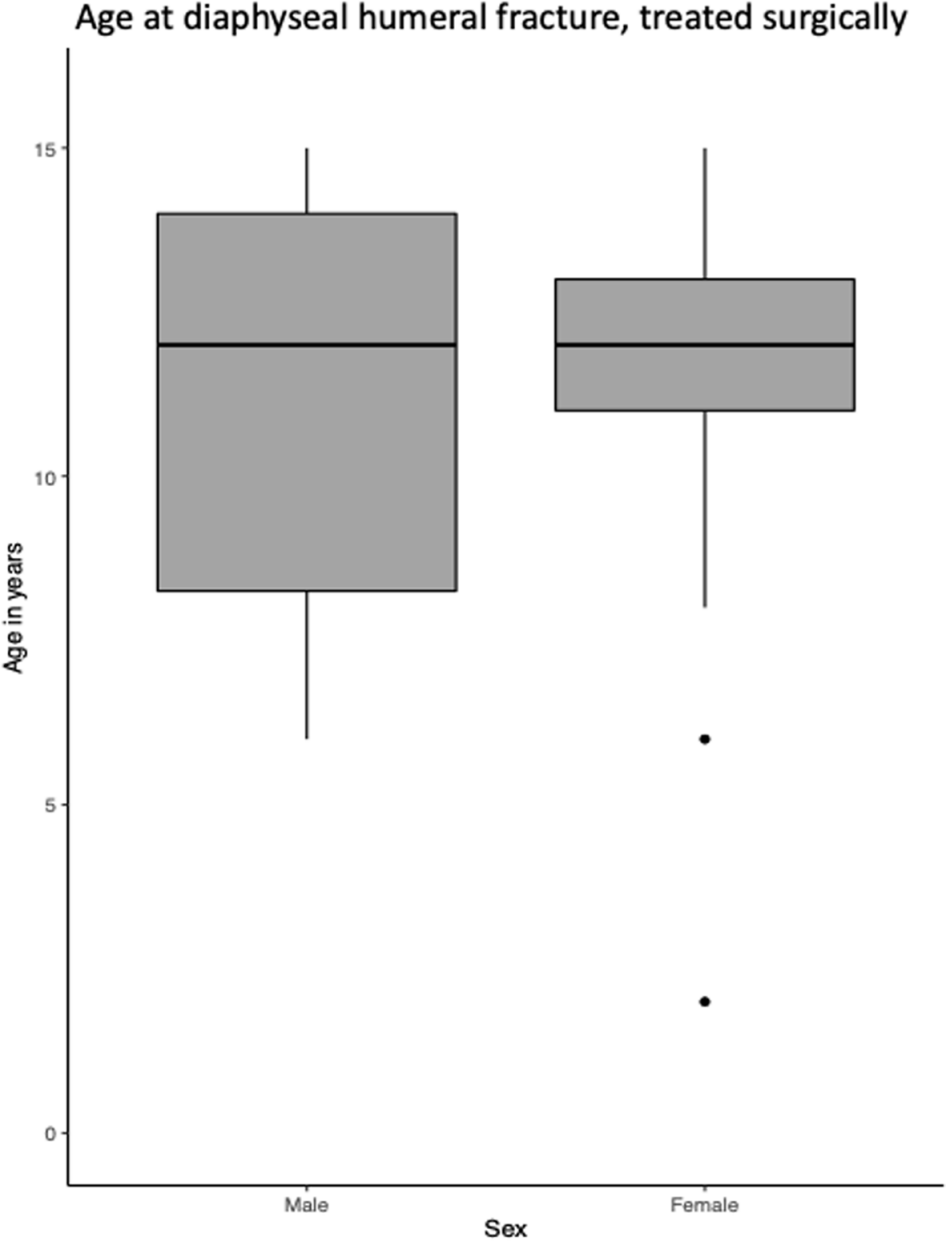
Age at surgical treated diaphyseal humeral fracture

In proximal fractures, the age at surgery was significantly lower among girls than boys (11 versus 12 years, *p* < 0.02). This was not the case in diaphyseal fracture (Figs. 9 and 10). 3 patients with proximal fractures and null patients with diaphyseal fracture had a registered reoperation.

99 proximal fractures were treated surgically. The preferred method in these fractures were K-wire and cerclage (65, 66%). 46 diaphyseal fractures were treated surgically. The preferred method in these fractures were intramedullary nailing (27, 59%). For all proximal and diaphyseal fractures, flexible nails were used in 46 (92%) of all 50 intramedullary nailing procedures (Tables 4 and 5).

**Table 4:** Treatment of proximal fractures in relation to patient’s sex and age

Proximal fractures											
	Non-surgical	Non-surgical	N-s with closed reduction	Surgical	Closed reduction under general anesthesia	K-wire and cerclage	Intramedullary nailing	Plate fixation	Screw fixation	Combined ostosynthesis	Not registered
**Girls, n (%)**											
**Age**											
0–3	41 (98)	41	0	0 (0)	0	0	0	0	0	0	1 (2)
4–6	139 (97)	138	1	1 (1)	0	1	0	0	0	0	3 (2)
7–9	247 (93)	244	3	12 (5)	0	9	3	0	0	0	7 (3)
10–12	387 (93)	384	3	26 (6)	2	14	6	1	1	1	5 (1)
13–15	119 (88)	119	0	14 (10)	1	11	1	1	0	0	3 (2)
Total	933 (93)	926	7	52 (5)	3	35	10	2	1	0	19 (2)
**Boys, n (%)**											
**Age**											
0–3	45 (94)	45	0	2 (4)	0	1	1	0	0	0	1 (2)
4–6	68 (93)	68	0	2 (3)	1	1	0	0	0	0	3 (4)
7–9	143 (95)	140	3	5 (3)	0	3	2	0	0	0	3 (2)
10–12	200 (92)	198	2	12 (6)	2	5	5	0	0	0	6 (3)
13–15	172 (85)	168	4	26 (13)	1	20	5	0	0	0	4 (2)
Total	628 (91)	619	9	47 (7)	4	30	13	0	0	0	17 (2)

**Table 5:** Treatment of diaphyseal fractures in relation to patient’s sex and age

Diaphyseal fractures											
	Non-surgical	Non-surgical	N-s with closed reduction	Surgical	Closed reduction under general anesthesia	K-wire and cerclage	Intramedullary nailing	Plate fixation	Screw fixation	Extern fixation	Not registered
**Girls, n (%)**											
**Age**											
0–3	15 (83)	15	0	3 (17)	2	0	0	1	0	0	0 (0)
4–6	15 (94)	15	0	1 (6)	0	0	1	0	0	0	0 (0)
7–9	18 (86)	17	1	1 (5)	0	0	1	0	0	0	2 (10)
10–12	20 (69)	19	1	5 (17)	0	0	5	0	0	0	4 (14)
13–15	13 (68)	13	0	5 (26)	0	1	2	2	0	0	1 (5)
Total	81 (79)	79	2	15 (15)	2	1	9	3	0	0	7 (7)
**Boys, n (%)**											
**Age**											
0–3	34 (100)	32	2	0 (0)	0	0	0	0	0	0	0 (0)
4–6	26 (96)	25	1	1 (4)	0	1	0	0	0	0	0 (0)
7–9	22 (69)	21	1	8 (25)	0	2	5	0	1	0	2 (6)
10–12	33 (79)	32	1	7 (17)	0	1	5	1	0	0	2 (5)
13–15	45 (73)	39	6	15 (24)	1	0	8	5	0	1	2 (3)
Total	160 (81)	149	11	31 (16)	1	4	18	6	1	1	6 (3)

## Discussion

### Main findings

21% of all humeral fractures were proximal and 4% were diaphyseal. Girls were overrepresented in patients with proximal fractures and boys in diaphyseal fractures. Surgical treatment methods increased with increasing age, but they were not associated with sex.

### Fracture epidemiology

As previous studies on general paediatric fracture epidemiology have reported a predominance in boys, we hypothesised that most humeral fractures would occur in boys. As opposed to this, we found that most fractures occurred in girls. This due to the female dominance in proximal fractures. Previous studies have reported a girl:boy ratio of 1.4:1 [12], 1.8:1 [16] and 9.5:1 [11] in proximal fractures. Proximal humeral fractures are one of few paediatric fractures mainly afflicting girls and might be explained by the female predominance in horse-riding [12]. These findings are in line with our findings with a majority of proximal fractures in girls being caused by horse-riding. Data from The Swedish Research Council for Sport Science show that 96% of all children participating in horse-riding are girls. On the contrary, 94% of all participating in ice-hockey are boys [17]. This sex-specific difference in sporting activities can explain the female peak incidence in May and male peak incidence in January. Our results on seasonal variation and mechanism of injury confirms the hypothesis that most children sustain their fracture during recreational activities.

The unimodal age distribution in proximal fractures with an earlier incidence peak in girls than boys was in line with other nationwide studies of paediatric fracture epidemiology [1, 7, 10]. The earlier peak in injuries amongst girls compared to boys could be explained by earlier puberty and growth spurt which causes a decrease in bone mineral density, making pubertal children more vulnerable to fractures [18]. In addition, our peak occurrences in diaphyseal fractures among infants and adolescents were also in line with other studies [5, 19]. The high incidence amongst infants can be explained by birth-related traumas, while traffic-related fractures might explain the increase in adolescents which is comparable with the cause of humeral fractures in adults [20].

### Fracture classification

To our knowledge, this is the first study that analysed the anatomical fracture classification in paediatric proximal and diaphyseal humeral fractures. Only few paediatric humeral fractures are multi-fragmentary fractures which is in contrast with the reports of adult fractures. Bergdahl et al. reported in 2016 that 55% of all proximal and 16% of all diaphyseal fractures are multi-fragmentary fractures in adults [20]. This could be explained by the biomechanical properties of paediatric bone and the occurrence of a growth plate in the affected area [21]. In adults the relation to osteoporosis and lower bone mineral density could explain their vulnerability to multi-fragmentary fractures [20, 22].

### Treatment

As previously described [23, 24], the most common treatment for humeral fractures was non-surgical treatment. In a Finnish study on proximal fractures, 8% were treated surgically [12] which corresponds well with our findings (7% in boys and 5% in girls). In another Finnish study on diaphyseal fractures, 50% of all hospitalised children required surgery and/or fracture reduction [19]. In our population 20% of all patients with diaphyseal fractures were treated surgically or had a fracture reduction. This reflecting our inclusion of both hospitalised and non-hospitalised patients.

Regarding the choice of surgical technique, there is an ongoing discussion on which surgical method should be used [25–28]. This varies both globally and in our study cohort. The diversity of surgical methods used indicates a more local tradition-based rather than evidence-based reason to choose one technique over the other. It might of course also reflect that the surgical technique chosen might - at least in children - not have such big impact on fracture healing since the reoperation rate is generally low. This also reflects the absence of randomised studies in children.

As hypothesised, we noted an increase in surgical treatment with increasing age. When considering surgery for the treatment of paediatric fractures, the child’s bone age plays an important role: in young children most fractures can be treated non-surgically due to open growth plates and substantial remodelling capacity [24]. However, with increasing age the remodelling capacity is decreasing and surgical treatment is more often required to achieve alignment [13, 14]. Furthermore, fracture healing-time increases with age [29]. Older children would therefore need longer immobilisation periods. Considering the fact that girls reach puberty earlier than boys, we would have expected that age at surgery was younger in girls. This was the case in proximal humeral fractures but not in diaphyseal fractures. The choice of treatment, non-surgical or surgical was however not associated with sex.

### Strengths and limitations

One notable strength of this study is the register design. The SFR is a nationwide register covering about 80% of Sweden’s orthopedic units at our time of study, stretching from small countryside hospitals to university hospitals. Another notable strength are the very low numbers of incomplete data. Only a few percent of the registered fractures lacked information on injury mechanism, classification or treatment. The fractures were classified by the treating orthopaedic surgeon and previous validation studies have reported good accuracy in such classifications [30–32].

As with all register-based studies, a prominent limitation is the lack of completeness when comparing registered fractures with fractures registered in the NPR. However, the NPR-data are less suitable for an epidemiological study since they lack detailed information on injury site, injury mechanism and fracture classification. Another limitation with register-based studies is their vulnerability to misinterpretation of the variables when registering a fracture. The fact that some units only register paediatric fractures treated surgically, can cause selection bias and a false high frequency of surgical treated fractures. Outcomes in the SFR include registrations of reoperations which is out of the scope of this epidemiological study. Furthermore, reoperations need verification of medical records to be fully valid.

## Conclusions

One in four paediatric humeral fractures occurred in the proximal or diaphyseal part. Girls were in majority in proximal fracturs and boys in diaphyseal fractures. Most children sustained their proximal and diaphyseal humeral fracture by falling from playground facilities, horse-riding or winter sport activities. Further studies need to investigate if these fractures could be prevented with prophylactic efforts in activities. The surgical treatment increased with increasing age in in both sexes but the age at surgery was younger in girls with proximal humeral fractures. The surgical method showed a large variation of techniques and the registered re-operation rate was very low.

## Data Availability

The dataset analysed in this study is not publicly available as the study was approved on the ground of ensuring the confidentiality of data of patients included in the study. We are positive to sharing data but are legally restricted to share the data publicly according to the law on Public Access and Secrecy, chapter 21, paragraph 7 and chapter 25, paragraph 1 (https://www.riksdagen.se/sv/dokument-lagar/dokument/svensk-forfattningssamling/offentlighets%2D%2Doch-sekretesslag-2009400_sfs-2009-400). Any person interested in the data set may contact Uppsala University and the corresponding author to find ways to share data according to Swedish laws and regulations. It is also possible for individuals interested in this data to apply directly from the Center of Registers, Västra Götaland (URL: http://registercentrum.se/). A process that involves an approval from the Swedish Ethical Review Authority.
